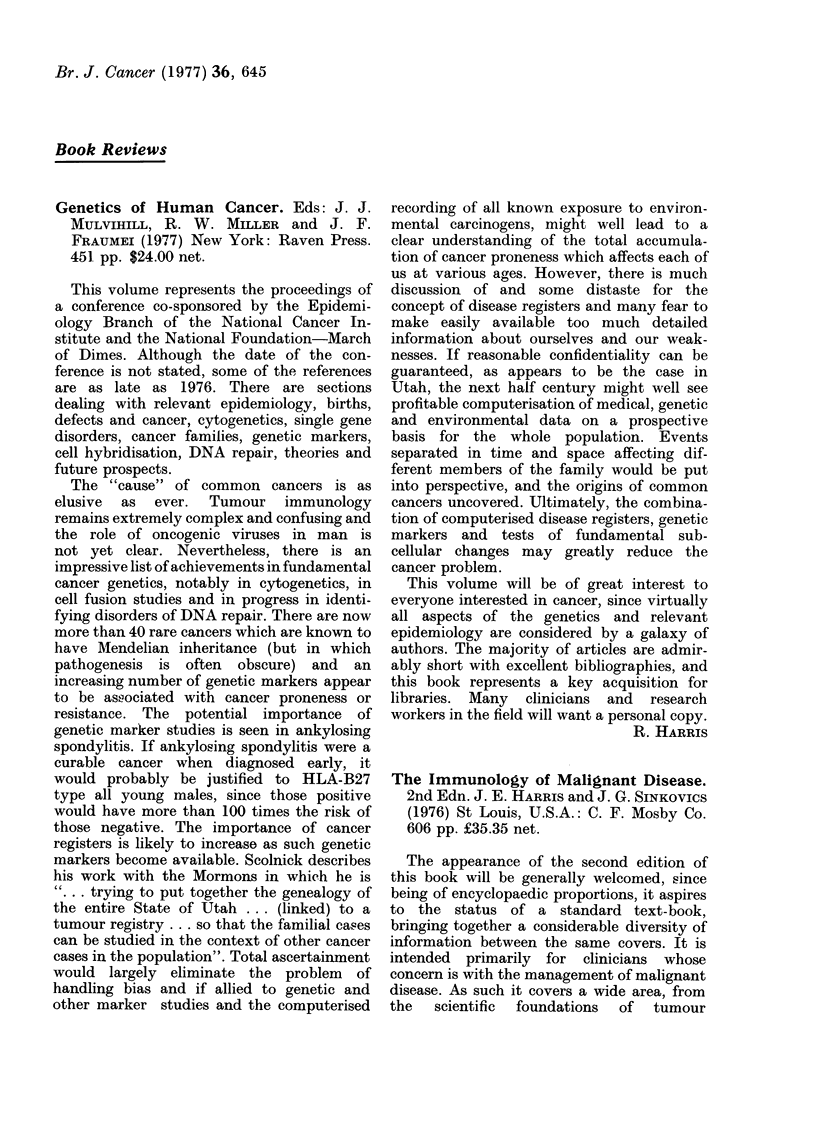# Genetics of Human Cancer

**Published:** 1977-11

**Authors:** R. Harris


*Br. J. Cancer* (1977) **36**, 645

### *Book Reviews*

**Genetics of Human Cancer.** Eds: J. J. MULVIHILL, R. W. MILLER and J. F. FRAUMEI (1977) New York: Raven Press. 451 pp. $24.00 net.

This volume represents the proceedings of a conference co-sponsored by the Epidemiology Branch of the National Cancer Institute and the National Foundation—March of Dimes. Although the date of the conference is not stated, some of the references are as late as 1976. There are sections dealing with relevant epidemiology, births, defects and cancer, cytogenetics, single gene disorders, cancer families, genetic markers, cell hybridisation, DNA repair, theories and future prospects.

The "cause" of common cancers is as elusive as ever. Tumour immunology remains extremely complex and confusing and the role of oncogenic viruses in man is not yet clear. Nevertheless, there is an impressive list of achievements in fundamental cancer genetics, notably in cytogenetics, in cell fusion studies and in progress in identifying disorders of DNA repair. There are now more than 40 rare cancers which are known to have Mendelian inheritance (but in which pathogenesis is often obscure) and an increasing number of genetic markers appear to be associated with cancer proneness or resistance. The potential importance of genetic marker studies is seen in ankylosing spondylitis. If ankylosing spondylitis were a curable cancer when diagnosed early, it would probably be justified to HLA-B27 type all young males, since those positive would have more than 100 times the risk of those negative. The importance of cancer registers is likely to increase as such genetic markers become available. Scolnick describes his work with the Mormons in which he is "... trying to put together the genealogy of the entire State of Utah ... (linked) to a tumour registry ... so that the familial cases can be studied in the context of other cancer cases in the population". Total ascertainment would largely eliminate the problem of handling bias and if allied to genetic and other marker studies and the computerised recording of all known exposure to environmental carcinogens, might well lead to a clear understanding of the total accumulation of cancer proneness which affects each of us at various ages. However, there is much discussion of and some distaste for the concept of disease registers and many fear to make easily available too much detailed information about ourselves and our weaknesses. If reasonable confidentiality can be guaranteed, as appears to be the case in Utah, the next half century might well see profitable computerisation of medical, genetic and environmental data on a prospective basis for the whole population. Events separated in time and space affecting different members of the family would be put into perspective, and the origins of common cancers uncovered. Ultimately, the combination of computerised disease registers, genetic markers and tests of fundamental subcellular changes may greatly reduce the cancer problem.

This volume will be of great interest to everyone interested in cancer, since virtually all aspects of the genetics and relevant epidemiology are considered by a galaxy of authors. The majority of articles are admirably short with excellent bibliographies, and this book represents a key acquisition for libraries. Many clinicians and research workers in the field will want a personal copy.

R. HARRIS